# Precise U-Pb age constrains on the Ediacaran biota in Podolia, East European Platform, Ukraine

**DOI:** 10.1038/s41598-018-38448-9

**Published:** 2019-02-08

**Authors:** Y. Soldatenko, A. El Albani, M. Ruzina, C. Fontaine, V. Nesterovsky, J.-L. Paquette, A. Meunier, M. Ovtcharova

**Affiliations:** 1 Université de Poitiers, Institut de Chimie des Milieux et Matériaux de Poitiers, CNRS UMR 7285, 86073 Poitiers, France; 2 Department of Geological Prospection, National Mining University of Ukraine, 49005 Dnipro, Ukraine; 3 Department of Geology, National Kiev University of Taras Shevschenko, Kyiv, Ukraine; 4 Laboratoire Magmas et Volcans, Université Clermont-Auvergne-CNRS-IRD-OPGC, 63000 Clermont-Ferrand, France; 5 Department of Earth Sciences, University of Geneva, 13 rue des Maraîchers, 1205 Geneve, Switzerland

## Abstract

The Neoproterozoic Era was characterized by rapidly changing paleogeography, global climate changes and especially by the rise and fall of the Ediacaran macro-biota. The correlation between disparate Ediacaran fossil-bearing localities and the tentative reconstruction of their paleoenvironmental and paleogeographic contexts are usually complicated by the lack of precise and accurate age data. For this reason, Neoproterozoic sedimentary sections associating Ediacaran biota fossils and fresh volcanic material are especially valuable for radioisotopic dating. Our research in the Podolya Basin, southwestern Ukraine, revealed the presence of four Neoproterozoic volcanic ash deposits (potassium-bentonite layers) within Ediacaran fossil-bearing siliciclastic rocks of the Mohyliv-Podilskyi Group. We used zircon U-Pb LA-ICPMS and CA-ID-TIMS methods to date two of those layers. The results indicate that a diverse assemblage of body and trace Ediacaran fossils occurred as early as 556.78 ± 0.18 million years (Ma) ago. By combining morphological evidence and new age determinations, we suggest a closer paleobiogeographical relationship between the Ukrainian Ediacaran assemblage and the Avalon paleocontinent than previously estimated.

## Introduction

The Neoproterozoic Era corresponds to a period of global changes related to the breakup of the supercontinent Rodinia and to protracted global glacial events^1^. In terms of biological evolution, it is associated with deep innovations likely related to the so-called ‘second great oxygenation event’ (NOE)^2^, and is marked by the rise and fall of the Ediacaran biota^3–8^. As revealed by over thirty sites inventoried worldwide^6,9–14^, the soft body imprints of the Ediacaran macro-organisms have been preserved in various marine environments and related deposits, such as carbonate rocks^15–17^, turbidites and volcanoclastic successions^18,19^, as well as siliciclastic deposits^20–22^.

Several species forming the Ediacaran biota - e.g., *Charnia* (575–545 Ma), *Dickinsonia* (560–541 Ma), *Onegia* (558–543 Ma), *Rangea* (558–545 Ma), *Palaeopascichnus* and *Tribrachidium* (558–541 Ma) - are long-lived taxa without substantial morphological change^10^, and their presence/absence thus does not represent a useful indicator for reliable biostratigraphical assessment. Additionally, the common lack of datable ash beds interlayered with the sedimentary sequences is the major obstacle for geochronological correlations between different Ediacaran fossil bearing sections^6,8,13^. In most contexts, the only way Ediacaran biostratigraphy could be appropriately placed into reliable chronological order is by high-precision radioisotopic dating of zircons from the products of large explosive volcanic eruptions such as ash, tuff or ignimbrite interlayered within Ediacaran fossil bearing strata^19,23–25^. In some Proterozoic terrains, ash deposits are altered and transformed into bentonite, whose chemical composition and mineralogy depend on the alteration processes and diagenetic history^26^.

In southwestern Ukraine, the siliciclastic deposits of the Mohyliv-Podilskyi Group outcropping in the Podolya Basin have revealed an abundant Ediacaran macrofauna^22^, but the preservation conditions of the fossil assemblages do not systematically grant secure biostratigraphic correlations at macro-regional scale across different sedimentary basins. For now, only one bentonite bed has been described in the Yarishyvska Formation^27^. However, the only available date for this context of 553 Ma^28^ is from the tuffaceous level without related information on its stratigraphical position as well as petrological description. Therefore, with the aim of constraining the chronological span of the Ediacaran fossils from the Podolya Basin, we have specifically investigated two among the four bentonite beds identified so far in the Mohyliv-Podilskyi Group.

By using LA-ICPMS and ID-TIMS dating methods, here we report U-Pb ages of two ash beds of the Mohyliv-Podilskyi Group interlayered with siliciclastic deposits where nearly the totality of the Ediacaran remains are embedded. Our results allow for the first time the integration of the paleobiological record from the Podolya Basin into a general Ediacaran biostratigraphic framework, which is a fundamental condition for more finely assessing the spatiotemporal relationships of the Ukrainian record with respect to the evidence from other Neoproterozoic basins in the context of the still debated paleogeographical location at that time of the paleocontinent Baltica^27^.

## Geological setting

**Figure 1:**
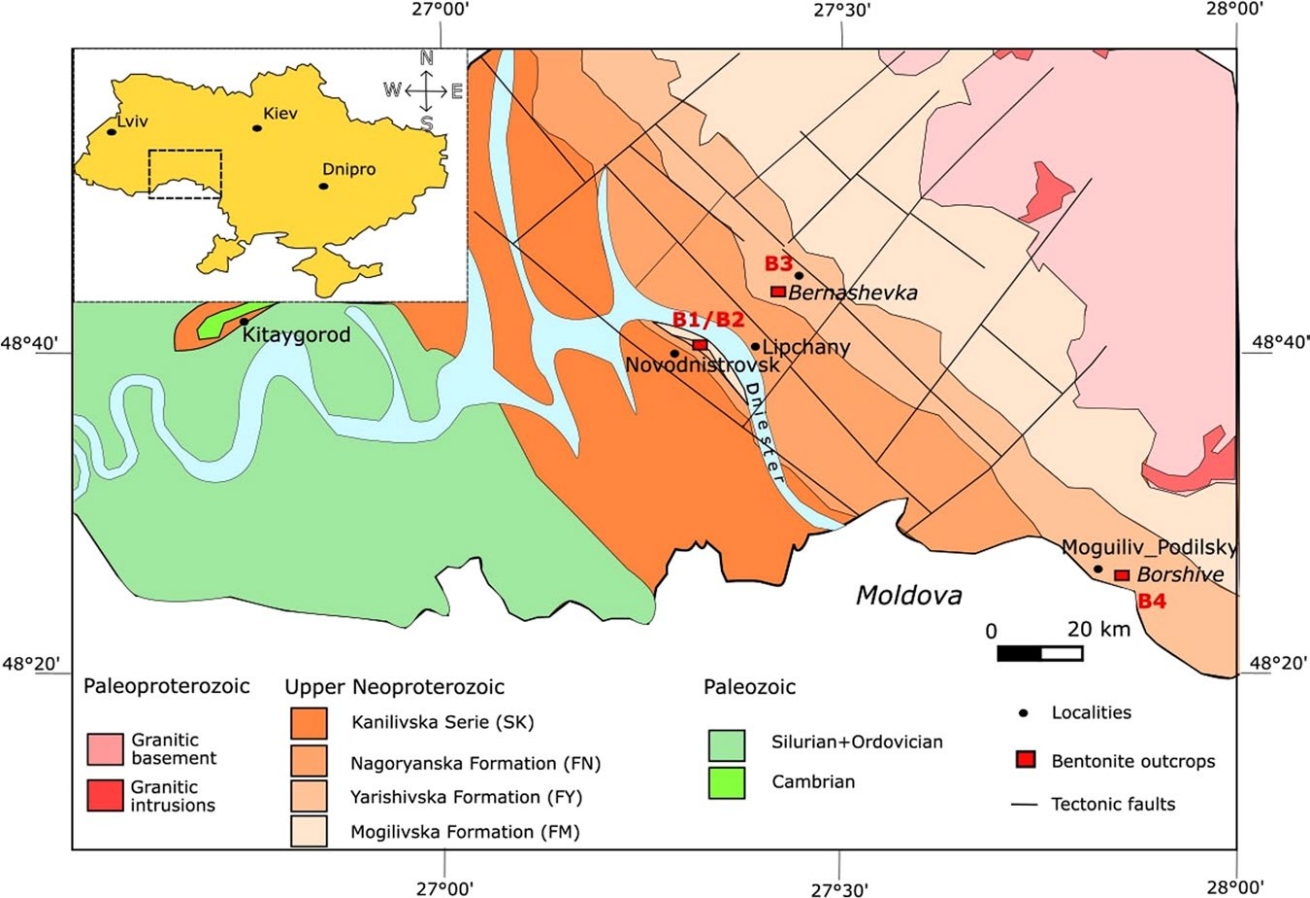
Synthetic geological map showing the relations between the Paleozoic-Precambrian sedimentary cover and the Archean basement rocks of the Ukrainian Shield and the position of the bentonite outcrops.

The Podolya Basin (Fig. 1) is located at the southwestern margin of the East European Platform, on the western flank of the Archean Ukrainian Shield^27,29^. It drains the hydrological basin of the Dniester River that cuts the Quaternary deposits overlaying the Neoproterozoic strata with an angular unconformity. In the studied area, the East European Platform is built on the continental shelf forming the edge of the Archean basement. The Neoproterozoic siliciclastic deposits represent the main part of the sedimentary column, while the considerably thinner Paleozoic (Cambrian-Silurian) strata are restricted to the southwestern part of the Podolya Basin. All these are unmetamorphic deposits.

**Figure 2:**
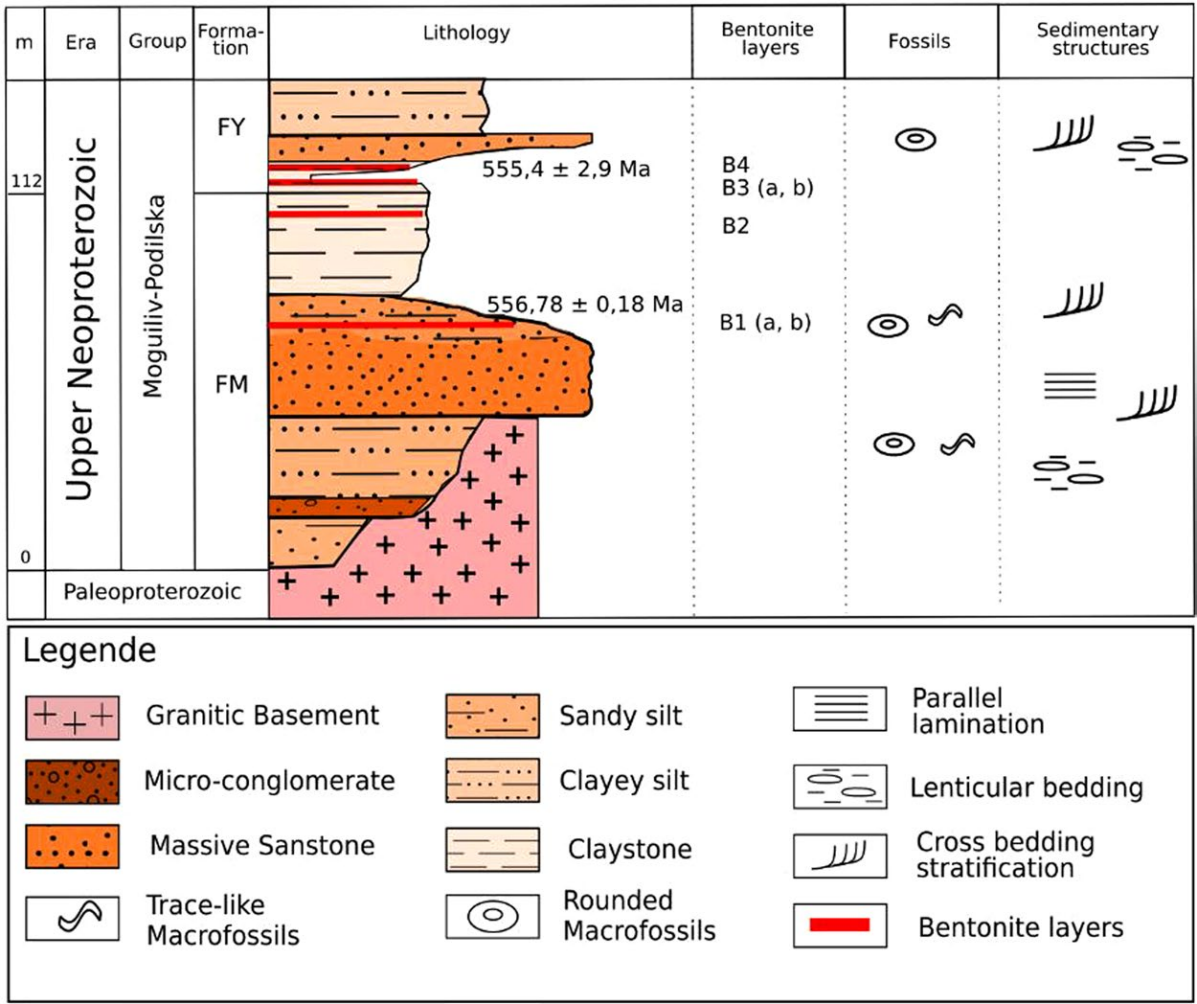
Stratigraphic Log (after^27–29^) showing a detailed lithostratigraphic column of the bottom part of the late Neoproterozoic of the southern Podolya Basin, southwestern Ukraine. FM: abbreviation for Mohylivska Formation; FY: abbreviation for Yarishyvska Formation.

The late Neoproterozoic sedimentary deposits of the Ukrainian Podolya Basin belong to the Mohyliv-Podilskyi and Kanilivska Groups. Imprints and fossil remains of representatives of the Ediacaran biota and bentonite beds are observed in the Mohylivska (FM) and Yarishyvska (FY) Formations forming the lower part of Mohyliv-Podilskyi Group (Fig. 2; a more detailed stratigraphic column of the late Neoproterozoic of the Podolya Basin is provided as Supplementary Information Fig. 1).

**Figure 3:**
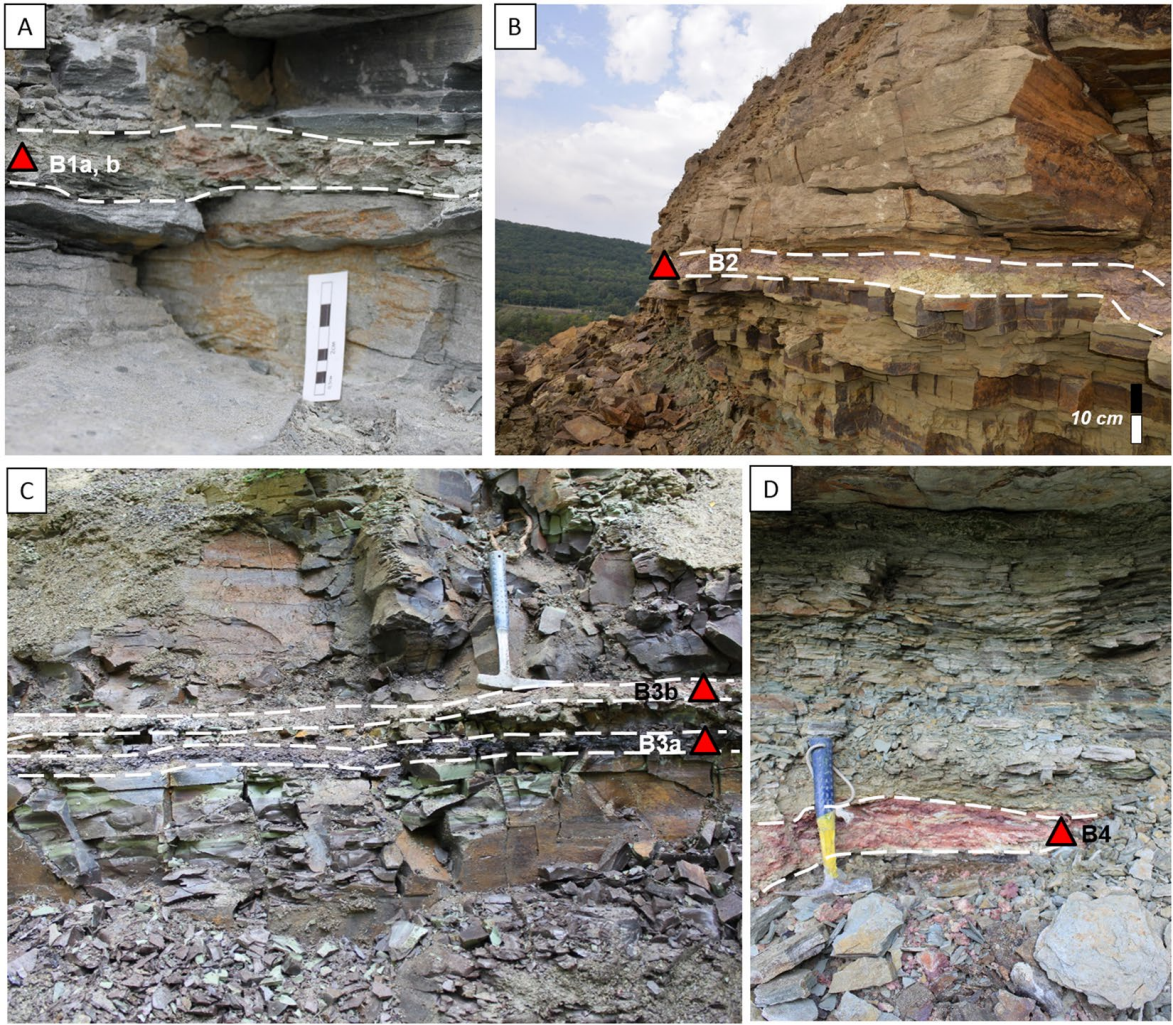
Field pictures of the bentonite beds occurring in FM (**A** and **B**) and FY (**C** and **D**) formations of the Podolya Basin. (**A**) bed B1 a (reddish) and b (greenish) from the Novodnistrovsky quarry with underlying sandstones including imprints of *Nemiana simplex* and overlying silty shales; the sample for zircon U-Pb CA-ID TIMS analyses is taken from bed B1 (greenish); (**B**) bed B2 from the Novodnistrovsky quarry with underlying silts and overlying massive sandstones; (**C**) bed B3 with two interbeds of bentonite - purple (a) and pink (b) - from the ravine near the Bernashevka village, with underlying and overlying massive silts; (**D**) bed B4 from the Borshiv ravine with underlying clayey silts and overlying sandy silts; the sample for zircon U-Pb LA-ICP-MS analyses is taken from bentonite B4.

**Figure 4:**
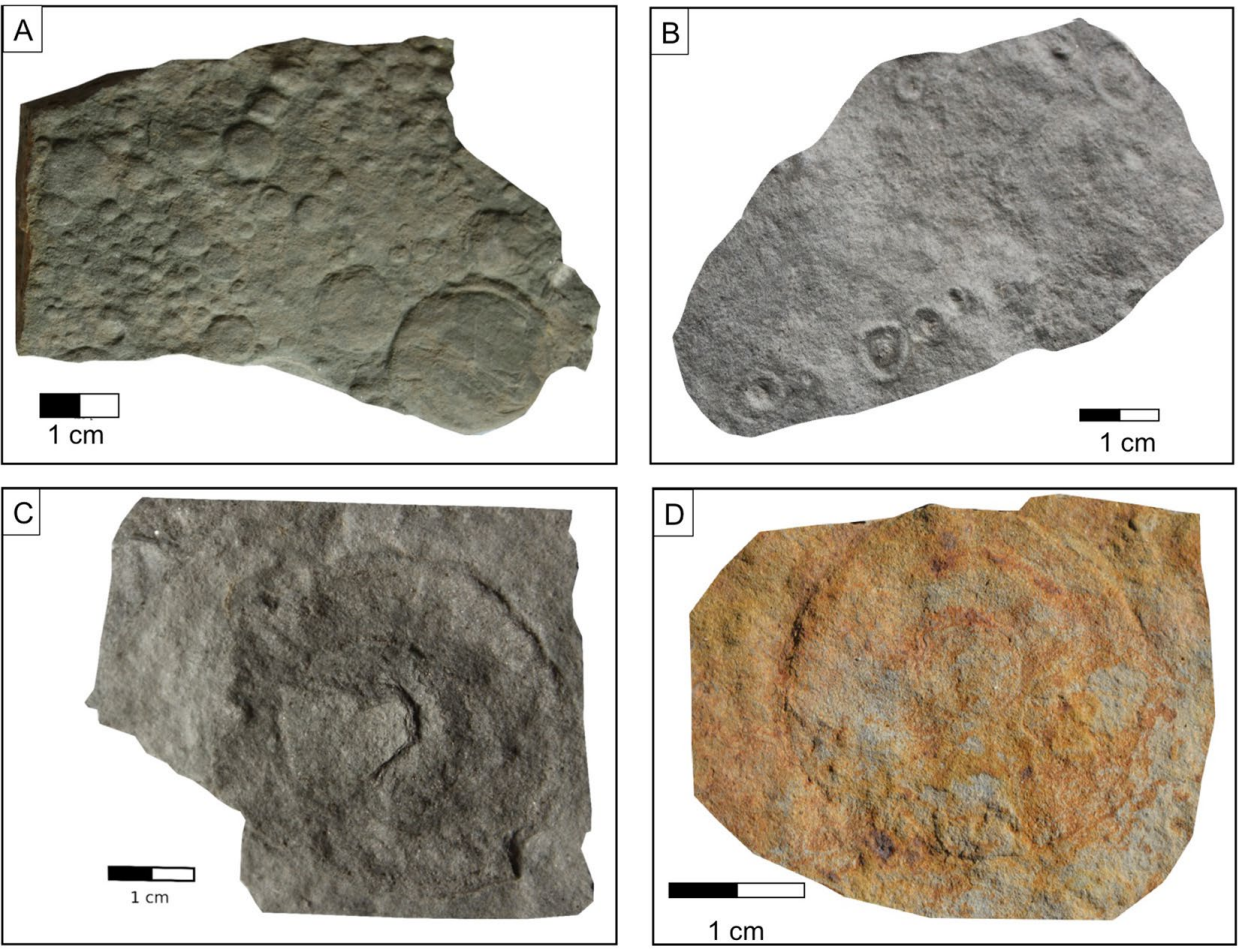
Ediacaran-type fossils from the Podolya Basin (morphotypes after^12,30^). (**A**) *Nemiana simplex* Palij; (**B**) *Intrites punctatus*; (**C**) *Gureevella elliptica* Menasova; (**D**) *Cyclomedusa plana* Glaessner and Wade.

In the lower part of the late Neoproterozoic sedimentary pile, at the transition between the two formations, four bentonite deposits (B1 to B4) have been identified (Figs 2, 3). Local examples of variably-sized Ediacaran macrofossils include *Gureevella elliptica, Intrites punctatus, Cyclomedusa plana* and *Nemiana simplex*^22,30^, ranging from a few millimeters to a few decimeters in diameter. Thin layers of microbial mats have preserved the discoidal forms in grouped (Fig. 4A,B) or isolated morphological units (Fig. 4C,D). Both “flinders-style” and “death mask-style” preservation forms are represented^31^. Discoidal forms and trace fossils have maximum abundance in the coarse-grained facies at the base of the sedimentary pile. Most of the soft-bodied imprints occur between the B1 and B2 bentonite levels of the Mohylivska Formation. The sandy level underlying the first level contains well-preserved imprints of *Nemiana simplex* (Fig. 3A). No evidence of Ediacaran fauna were found above and below the second dated B4 layer (Fig. 3D). At the Mohylivska-Yarishyvska transition, the facies deposits change from sandstone (upper part of FM - V2lyad sequence) to clayey siltstone (basal part of overlying FY - V2bern) (Fig. 2). Except for some rare rounded macrofossils observed immediately above the B4-layer, the Ediacaran-type forms disappear in the upper silty-shaly sediments of the section.

## Results

### Lithostratigraphy

From the bottom of the Mohylivska (FM) to the base of the Yarishyvska (FY) Formations, lithofacies are fine-to-medium grained (clayey silts to massive sandstone facies), whereas the transition from FM to FY is more clayey^22,27,29^.

Four bentonite beds were sampled in three locations: the two lower ones, B1 and B2, are associated with the top part of FM in the Novodnistrovsky quarry (48°3′N, 27°2′E) - B1 (a, b) and B2 beds (Fig. 3A,B). The two upper levels, B3 and B4, which correspond to the base of FY, were discovered in a ravine near the locality of Bernashevka (48°1′N, 27°1′E) - B3 (a, b) bed (Fig. 3C), and in the Borshive ravine near the city of Moguilive-Podilsky (48°1′N, 27°2′E) - B4 bed (Fig. 3D), respectively. The bentonite beds were clearly identified due to their clayey character and bright color^26^ which distinctly contrasts with the generally grey to greenish-gray sediments in which they are intercalated.

### Geochemistry

**Figure 5:**
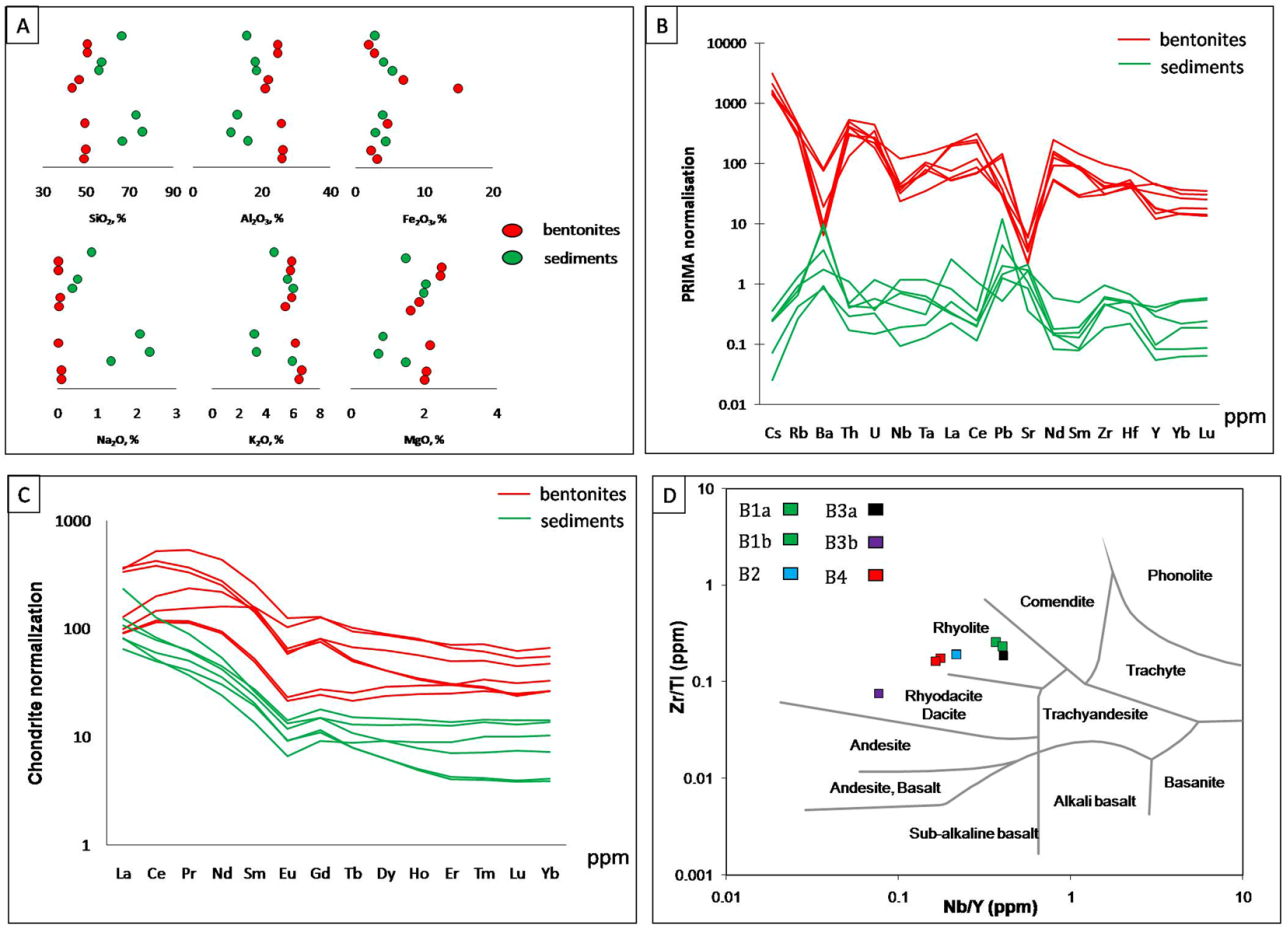
(**A**) Comparison of major elements content in lower and upper siliciclastic sediments vs. intercalated bentonite beds in FM and FY Formations of the Podolya basin; (**B**) spidergrams distribution of trace elements content after primitive mantle normalization; (**C**) rare earth elements (REE) patterns after chondrite normalization for bentonites and host rocks; (**D**) positioning of the bentonite beds in the rhyolitic domain of Nb/Y - Zr/Ti diagram used to delimit volcanic rock fields (from^40^).

Compared to the immediately below and above siliciclastic sediments (Fig. 5A), the major elemental composition of all four bentonite beds show important differences (Fig. 5A). According to the absence of detrital quartz and feldspars, the contents in SiO_2_ and Na_2_O are less than 51% and nearly zero, respectively, which is much lower than those of their host sediments (Table 1). On the contrary, the contents in Al_2_O_3_ (>24%) and MgO (>1.65%) are higher because of the high abundance of clays. Small amounts of iron can be incorporated in the crystalline lattice of I:S clay minerals in the bentonite, but most of it is contained in iron-bearing phases, such as hematite or poorly crystallized oxides, which indicate oxidizing conditions during alteration of primary volcanic ash^32^. Indeed, the iron content in the bentonite beds varies from 1.86% to 6.98%, except for an anomaly of 14.99% in the B3 (a) sample. The bentonite content in K_2_O is lower than those recorded in the host siliciclastic rocks, which in turn contain detrital muscovite and K-feldspar. None of these inherited minerals are present in the bentonite, where the potassium is exclusively related to illite/smectite mixed-layer minerals (Supplementary Fig. 2). The chemical and mineralogical compositions of each bentonite level are hence typical of K-bentonites^33^.

**Table 1:** Chemical composition of the K-Bentonite beds with lower and upper siliciclastic sediments.

%	Bentonites							Siliciclastic sediments					
	B1a	B1b	B2	B3a	B3b	B4a	B4b	FM3	FM5	FM8	FY1	FY3	FY6
**SiO**_**2**_	49,17	50,12	49,33	43,44	46,96	50,44	50,45	66,84	76,11	72,92	55,76	57,37	66,69
**Al**_**2**_ **O**_**3**_	25,95	26,23	25,79	21,10	22,06	24,71	24,74	16,00	11,07	12,90	18,51	18,23	15,72
**Fe**_**2**_ **O**_**3**_	3,13	2,22	4,65	14,99	6,98	2,74	1,86	4,39	2,92	3,99	5,38	4,10	2,83
**MnO**	0,00	0,00	0,02	0,05	0,04	0,02	0,02	0,03	0,04	0,02	0,02	0,02	0,03
**MgO**	2,04	2,08	2,19	1,65	1,89	2,46	2,50	1,51	0,76	0,89	2,00	2,06	1,51
**CaO**	0,62	0,66	0,43	0,74	2,63	0,89	0,87	0,47	1,20	0,38	0,15	0,10	0,67
**Na**_**2**_ **O**	0,10	0,11	0,03	0,05	0,07	0,03	0,03	1,35	2,33	2,08	0,37	0,51	0,86
**K**_**2**_ **O**	6,46	6,68	6,20	5,43	5,93	5,84	5,92	5,96	3,29	3,13	6,04	5,60	4,62
**TiO**_**2**_	0,22	0,24	0,27	0,85	1,04	0,39	0,39	0,62	0,31	0,44	1,28	1,25	0,63
**P**_**2**_ **O**_**5**_	0,00	0,00	0,00	0,14	1,40	0,00	0,00	0,00	0,00	<L.D.	0,11	<L.D.	0,00
**PF**	12,49	11,74	11,69	11,31	11,68	12,36	12,84	3,47	3,23	3,00	10,62	10,37	7,01
**Total**	100,18	100,08	100,59	99,75	100,66	99,88	99,62	100,64	101,26	99,74	100,24	99,61	100,56
**ppm**													
**Rb**	344,50	351,27	359,63	248,38	311,88	213,01	218,04	228,41	95,42	105,42	289,50	279,50	170,81
**Cs**	30,70	32,79	69,17	31,39	46,25	36,16	34,52	7,73	1,75	2,23	11,62	13,04	8,87
**Be**	7,89	9,05	2,43	2,73	2,55	1,80	1,72	4,14	1,96	1,37	4,32	4,92	3,51
**Ba**	62,06	69,09	592,28	551,78	140,16	69,11	46,60	681,86	550,75	463,86	240,71	249,67	413,59
**Sr**	77,41	79,40	66,34	113,87	121,18	45,11	41,91	131,25	113,08	96,38	43,44	48,43	88,76
**Zr**	305,18	378,18	308,29	951,53	473,10	385,64	414,05	357,98	140,69	177,47	210,23	233,85	237,40
**Hf**	11,95	14,71	11,07	21,97	11,47	12,71	13,33	9,85	3,49	4,84	5,90	6,40	6,56
**U**	4,34	5,97	10,51	6,15	8,29	6,56	5,26	3,36	1,54	2,00	3,06	7,69	2,10
**Th**	39,83	48,70	52,39	40,45	12,85	29,01	30,91	19,35	8,91	11,86	13,90	13,77	13,36
**V**	17,55	20,76	6,94	52,12	50,94	22,52	18,87	66,09	39,31	48,86	184,37	210,59	92,81
**Nb**	20,34	22,52	28,72	75,39	14,74	25,64	26,39	9,24	5,44	6,95	17,36	19,21	18,46
**Ta**	2,87	3,40	3,81	5,41	1,25	2,50	2,58	1,04	0,80	0,69	1,46	1,56	1,40
**Cr**	5,02	3,19	1,63	6,90	2,93	17,02	22,17	68,20	46,22	67,23	123,05	154,36	67,86
**Mo**	<L.D.	<L.D.	<L.D.	0,53	<L.D.	0,00	0,00	0,00	0,00	<L.D.	3,52	1,76	0,00
**W**	0,98	<L.D.	3,32	3,21	3,99	0,00	0,00	1,17	0,00	<L.D.	2,21	2,36	1,19
**Co**	9,35	12,70	2,68	16,59	6,32	0,99	1,61	6,12	1,98	6,39	25,18	11,33	3,05
**Ni**	15,71	23,19	4,87	18,06	12,90	7,09	6,92	24,00	13,74	18,65	25,92	19,75	10,08
**Cu**	3,43	5,05	2,68	82,32	86,24	0,00	2,06	12,29	5,77	4,61	111,73	31,96	8,86
**Zn**	7,66	8,09	15,45	21,85	22,39	35,80	39,47	37,99	13,58	14,40	168,41	50,88	25,39
**Cd**	0,06	0,07	0,05	0,14	0,08	0,13	0,11	0,06	0,03	0,02	0,06	0,04	0,07
**In**	0,11	0,13	0,11	0,10	0,09	0,06	0,06	0,05	0,00	<L.D.	0,10	0,09	0,06
**Ga**	33,55	34,54	38,02	23,70	24,80	27,90	28,28	28,06	15,80	14,44	28,97	30,72	23,74
**Pb**	22,63	25,25	5,20	9,82	5,28	4,97	6,54	12,83	10,33	12,35	63,26	21,82	9,52
**Sn**	7,87	8,17	12,12	6,72	3,57	6,58	6,40	3,50	1,53	2,28	3,90	4,08	2,97
**Ge**	1,89	1,57	2,61	4,54	2,27	2,00	1,73	1,74	1,35	1,51	2,17	2,32	1,64
**As**	3,78	7,96	0,54	1,62	0,87	1,16	3,91	0,51	0,55	2,98	15,76	31,03	2,53
**Sb**	0,91	1,08	0,17	0,61	0,34	0,16	0,16	0,00	0,00	0,11	2,06	1,95	0,13
**Bi**	0,57	0,80	0,68	0,42	0,08	0,65	0,52	0,47	0,24	0,14	0,46	0,37	0,26
**Sc**	19,56	22,37	13,78	22,52	25,87	14,57	15,00	11,08	6,04	9,00	30,16	25,12	13,46
**Y**	50,48	61,18	132,44	184,95	191,79	73,62	75,36	17,59	11,08	10,02	18,48	25,64	31,23
**La**	33,17	33,66	47,06	130,44	36,36	134,24	123,60	85,38	23,69	29,56	29,76	45,32	39,35
**Ce**	109,87	112,91	190,54	498,35	139,79	402,38	365,30	120,85	47,38	57,21	49,39	78,91	74,94
**Pr**	15,53	15,95	32,24	72,95	21,14	49,69	45,34	12,20	5,65	6,91	5,06	8,44	8,60
**Nd**	64,88	66,69	154,80	308,49	114,34	195,45	178,60	38,31	21,79	25,28	17,01	29,74	32,06
**Sm**	11,43	12,09	34,53	59,47	36,80	35,72	32,93	5,97	4,47	4,72	3,10	5,53	6,33
**Eu**	1,87	2,02	5,03	10,89	8,97	5,72	5,32	1,15	0,80	0,80	0,57	1,03	1,23
**Gd**	7,47	8,37	24,65	38,99	39,09	24,50	22,92	4,53	3,35	3,50	2,79	4,54	5,43
**Tb**	1,25	1,48	3,85	5,43	5,93	2,99	2,89	0,62	0,46	0,46	0,51	0,75	0,87
**Dy**	9,07	10,99	23,82	32,92	34,22	15,64	15,51	3,48	2,40	2,38	3,46	4,89	5,61
**Ho**	2,11	2,54	4,77	6,68	6,87	2,89	2,94	0,66	0,42	0,42	0,76	1,10	1,21
**Er**	6,27	7,53	12,33	17,60	16,57	7,50	7,65	1,74	1,01	1,05	2,22	3,14	3,38
**Tm**	0,94	1,20	1,78	2,53	2,20	1,01	1,03	0,25	0,14	0,15	0,35	0,48	0,51
**Lu**	0,96	1,20	1,70	2,36	2,04	0,91	0,91	0,28	0,15	0,15	0,38	0,49	0,54
**Yb**	6,60	8,18	11,72	16,31	13,78	6,54	6,52	1,78	0,96	1,01	2,56	3,36	3,49
**∑REE**	271,43	284,82	548,82	1203,41	478,09	885,18	811,46	277,20	112,67	133,57	117,93	187,71	183,55

Compared to their host sediments, the alkaline earth metals distribution (Table 1) of the bentonite beds normalized to primitive mantle (PRIMA)^34^ exhibit a systematic depletion of Ba and Sr contents related to the scarcity of micas and feldspars. These two elements are concentrated in the lower and upper sedimentary deposits where their mineral phases form the bulk of the detrital input. In contrast, Cs content is higher in bentonite material because this immobile element^33^ is easily absorbed by the newly formed clay minerals. Likewise, other elements, such as Nb, Ta, Zr, Hf and REEs (especially La, Ce, Nd, Sm and Y), which were immobile during the surficial alteration processes of volcanic ashes^35,36^, are enriched in bentonite products^37^. The slight Nb-Ta negative anomalies in bentonites are indicative of their volcanic origin in subduction setting.

The normalized chondrite^38^ REE spectra of bentonite beds and host sediments are significantly different (Fig. 5C). Average ∑REE values (1960 ppm), Y contents (73 ppm) and ∑LREE/∑HREE ratios (5,2) are, respectively, 6, 3 and 2 times higher than those of the silty sediments in the same units. Therefore, bentonite beds exhibit a specific geochemical signature. The absence of a positive Ce anomaly (at most concentrated REE, 420 ppm), is probably an indication of suboxic water conditions^39^ during ash alteration. On the other hand, the Eu depletion in bentonite is indicative of plagioclase fractionation in a magmatic source, while in the host sediments plagioclase is absent due to its sensitivity to weathering before sedimentation. Immobile components of the composition in each bentonite bed were plotted in a Nb/Y - Zr/Ti diagram^40^ and used to discriminate the compositional fields of common volcanic rocks (Fig. 5D). They all plot into the rhyolite or rhyodacite fields, an evidence which confirms trace elements distribution previously observed for this material (Fig. 5B) and typical of rhyolitic material^41,42^.

### Geochronology

The geochemical analyses show that B4 K-bentonite is almost exclusively illite/smectite mixed-layer without inherited minerals (Supplementary Fig. 2). This indicates no reworking and *in situ* ash-bentonite transformation. In these conditions, U-bearing minerals can be confidently used for absolute dating this volcanoclastic deposits. The correlative high concentrations of Zr and U suggest that zircon is the main carrier of uranium in B4 K-bentonite.

**Figure 6:**
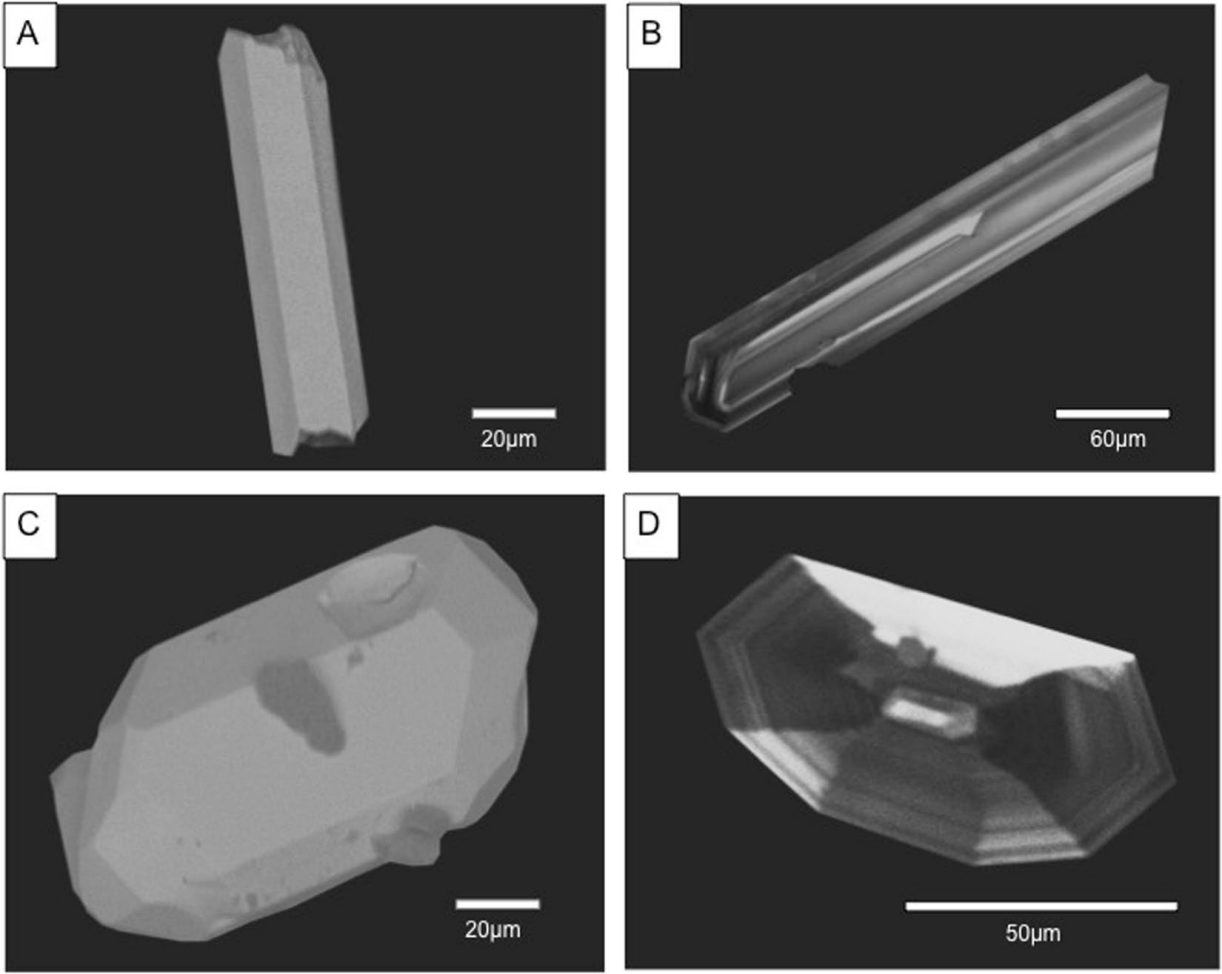
Morphological features of zircon crystals from the B4 bentonite bed.

Zircon crystals from the granular fraction (<4.5% weight) have a maximum size from 50 to 80 µm (Fig. 6). They are characterized by three typologies: mainly elongated acicular, euhedral, and subhedral. Regardless of shape, sharp edges indicate the absence of corrosion and transport. The needle-shaped acicular zircon crystals (Fig. 6A,B) indicate rapid zircon crystallization, while euhedral crystals exhibit some vesicles (Fig. 6C,D) possibly interpreted as fluid inclusions^43^ and/or gas bubbles^44^ trapped in their crystalline lattice.

**Figure 7:**
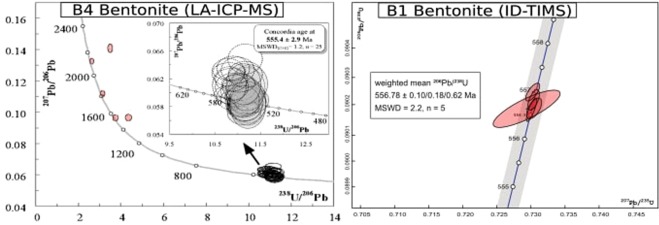
Concordia diagrams for U-Pb zircon dating of bentonites in the Podolia Basin. (**A**) Terra-Wasserburg concordia diagram showing the U-Pb age on the dated B4 bentonite sample. Analyses acquired using LA-ICP-MS; (**B**) concordia plot showing the result of high precision CA-ID-TIMS dating of zircons from the B1b bentonite sample NOV-ccg. All single error ellipsis are in 2σ; the age is given as weighted mean ^206^Pb/^238^U age within 2σ x/y/z error systematics^67^.

While needle-shaped acicular zircons are weakly zoned, other subhedral zircons display very regular fine-scale oscillatory zoning without solid inclusions. Moreover, complex growth associated to superimposed or disrupted zonings, or local recrystallizations, were never observed. Consequently, we suggest that these zircons crystallized within one episode, without zircon reworking from previous magmatic products. A total set of forty-three zircon crystals from bentonite B4 were analyzed by LA-ICP-MS and plotted in a Tera-Wasserburg ^238^U/^206^Pb vs. ^207^Pb/^206^Pb diagram^45^ (Fig. 7). Twenty-five concordant analyses yield a Concordia age of 555.4 ± 2.9 Ma (MSWD(C + E) = 1.2) (Table 2). Five zircon crystals revealed 1.3–2.2 Ga Meso and Paleoproterozoic inherited ages. The occurrence of such old zircons inherited in the magma chamber or scavenged from the basement during the volcanic eruption is frequently recorded^46^. The remaining three analysed zircons were not considered in the calculations owing to a small common Pb contribution. We interpret this age of 555.4 ± 2.9 Ma as dating the crystallization of the volcanic zircons.

**Table 2:** Zircon U-Pb data obtained by *in situ* Laser Ablation ICP-MS. 1: concentration uncertainty c. 20%; 2: data not corrected for common-Pb.

Analysis	*Pb*	*Th*	*U*	Th/U	^207^Pb/^235^U^2^	2 s error	^206^Pb/^238^U^2^	2 s error	Rho	Age (Ma)	2 s error	Age (Ma)	2 s error	Age (Ma)	2 s error	Concordancy^3^
	ppm^1^	ppm^1^	ppm^1^			^207^Pb/^235^U		^206^Pb/^238^U		207Pb/206Pb	207Pb/206Pb	207Pb/235U	207Pb/235U	^206^Pb/^238^U	^206^Pb/^238^U	%
z01	30	212	284	0,75	0,7432	0,0236	0,09107	0,00249	0,86	574	75	564	14	562	15	98
z02	23	151	218	0,69	0,7600	0,0250	0,09233	0,00252	0,83	593	75	574	14	569	15	96
z03	10	90	91	0,98	0,7718	0,0293	0,09210	0,00255	0,73	631	87	581	17	568	15	90
z04	29	173	281	0,61	0,8041	0,0256	0,09257	0,00252	0,86	708	73	599	14	571	15	81
z05	20	101	204	0,50	0,7464	0,0240	0,08995	0,00246	0,85	610	75	566	14	555	14	91
z06	23	144	227	0,63	0,7680	0,0245	0,09089	0,00246	0,85	649	74	579	14	561	15	86
z07	8,4	35	85	0,41	0,8041	0,0305	0,09276	0,00255	0,72	704	86	599	17	572	15	81
z08	13	126	114	1,10	0,7371	0,0265	0,08922	0,00243	0,76	600	83	561	15	551	14	92
z09	15	118	136	0,86	0,7502	0,0279	0,09183	0,00252	0,74	576	86	568	16	566	15	98
z10	8,8	68	81	0,84	0,7680	0,0305	0,09276	0,00255	0,69	605	91	579	18	572	15	95
z11	11	104	100	1,04	0,7058	0,0263	0,08979	0,00246	0,74	491	89	542	16	554	15	113
z12	13	124	109	1,14	0,7933	0,0290	0,09100	0,00249	0,75	716	83	593	16	561	15	78
z13	7,8	48	76	0,63	0,7784	0,0357	0,09254	0,00261	0,62	639	104	585	20	571	15	89
z14	20	199	174	1,14	0,7853	0,0274	0,09217	0,00249	0,77	667	81	589	16	568	15	85
z15	15	107	141	0,76	0,8020	0,0287	0,09182	0,00249	0,76	720	82	598	16	566	15	79
z16	18	283	146	1,94	0,7491	0,0311	0,09011	0,00249	0,67	614	95	568	18	556	15	91
z17	13	154	110	1,41	0,7221	0,0260	0,08986	0,00243	0,75	540	87	552	15	555	14	103
z18	11	87	107	0,81	0,7558	0,0266	0,09104	0,00246	0,77	611	82	572	15	562	15	92
z19	6,7	41	68	0,60	0,7494	0,0309	0,09009	0,00249	0,67	615	95	568	18	556	15	90
z20	15	84	155	0,54	0,7385	0,0251	0,09032	0,00243	0,79	578	80	562	15	557	14	96
z21	11	90	99	0,91	0,7356	0,0279	0,08996	0,00246	0,72	578	88	560	16	555	14	96
z22	25	341	193	1,77	0,7166	0,0235	0,09009	0,00240	0,81	518	79	549	14	556	14	107
z23	14	182	117	1,55	0,7203	0,0252	0,08931	0,00240	0,77	548	83	551	15	551	14	101
z24	14	140	128	1,10	0,7173	0,0304	0,08960	0,00246	0,65	532	100	549	18	553	15	104
z25	12	100	100	1,00	0,7193	0,0308	0,08969	0,00252	0,66	535	100	550	18	554	15	103
z26	13	92	122	0,75	0,7187	0,0278	0,08853	0,00246	0,72	562	91	565	16	547	15	97
z27	8,5	40	84	0,48	0,7446	0,0385	0,08930	0,00261	0,57	620	116	568	22	551	15	89
z28	6,5	29	66	0,45	0,7498	0,0351	0,08882	0,00255	0,61	647	105	551	20	549	15	85
z29	5,4	51	43	1,20	0,7206	0,0403	0,08915	0,00264	0,53	553	125	560	24	551	16	100
z30	6,7	60	55	1,10	0,7352	0,0344	0,08972	0,00258	0,61	583	106	598	20	554	15	95
z31	13	96	107	0,90	0,8012	0,0312	0,08965	0,00249	0,71	768	87	574	18	554	15	72
z32	12	49	124	0,39	0,7599	0,0296	0,09064	0,00252	0,71	632	89	552	17	559	15	88
z33	6,4	44	58	0,75	0,7224	0,0334	0,08955	0,00255	0,62	549	106	548	20	553	15	101
z34	6,0	61	47	1,31	0,7162	0,0344	0,08912	0,00255	0,60	540	112	548	20	550	15	102
z35	4,0	32	37	0,87	0,7149	0,0401	0,08914	0,00261	0,52	535	129	557	24	551	15	103
z36	10	72	99	0,73	0,7299	0,0307	0,09013	0,00252	0,66	557	97	582	18	556	15	100
z37	30	101	323	0,31	0,7733	0,0286	0,08963	0,00246	0,74	694	85	582	16	553	14	80
z38	12	48	123	0,39	0,7583	0,0371	0,08990	0,00255	0,58	645	110	573	21	555	15	86
z39	38	111	117	0,95	3,5359	0,1139	0,2669	0,007200	0,84	1549	66	1535	26	1525	37	98
z40	38	103	145	0,71	3,0549	0,0966	0,2303	0,006180	0,85	1552	65	1422	24	1336	32	86
z41	108	157	254	0,62	6,9556	0,2005	0,3811	0,010140	0,92	2129	56	2106	26	2082	47	98
z42	61	126	167	0,76	4,8986	0,1499	0,3188	0,008550	0,88	1823	61	1802	26	1784	42	98
z43	69	159	221	0,72	5,5384	0,1736	0,2858	0,007740	0,86	2234	59	1907	27	1621	39	73

a - Th contents calculated from radiogenic ^208^Pb and ^230^Th-corrected ^206^Pb/^238^U date of the sample, assuming concordance between U-Pb Th-Pb systems; b - Total mass of radiogenic Pb; c - Total mass of common Pb; d - Ratio of radiogenic Pb (including ^208^Pb) to common Pb; e - Measured ratio corrected for fractionation and spike contribution only; f - Measured ratios corrected for fractionation, tracer and blank; g - Isotopic dates calculated using λ238 = 1.55125E-10 (Jaffey et al. 1971) and λ235 = 9.8485E-10 (Jaffey et al. 1971); h - Corrected for initial Th/U disequilibrium using radiogenic ^208^Pb and Th/U[magma] = 3.50000. ^1^concentration uncertainty c. 20%, ^2^data not corrected for common-Pb, ^3^(^206^Pb/^238^U age)/(^207^Pb/^206^Pb age) * 100.

Zircons from bentonite B1 bed was analysed by CA-ID-TIMS method. All data are interpreted in terms of weighted mean ^206^Pb/^238^U dates by defining the youngest coherent population which yields statistically acceptable MSWD (Table 3). The highest temperature of chemical abrasion (210 °C) and a 13 h duration have been proven to eliminate the residual lead loss effect in the zircon crystals. Accordingly, we are confident that the reported weighted mean dates represent the age of the ash deposition.

**Table 3:** Zircon U-Pb data obtained by the ID-TIMS method.

Analysis	Composition				Isotopic Ratios							Corr.	Dates (Ma)								% disc.
	Th/	Pb*	Pbc	Pb*/	206Pb/	207Pb/	±2σ %	207Pb/	±2σ %	206Pb/	±2σ %		207Pb/	±2σ	207Pb/	±2σ	206Pb/	±2σ	206Pb/	±2σ	
																			238U		
	U a	(pg) b	(pg) c	Pbc d	204Pb e	206Pb f		235U f		238U f		coef.	206Pb g	abs	235U g	abs	238U g	abs	<Th>h	abs	
_z2	0.56	12.6	0.18	69	3887	0.05877	0.62	0.7303	0.67	0.090171	0.073	0.813	558	13	556.8	2.9	556.55	0.39	556.63	0.39	0.19
_z3	0.60	53.2	0.18	299	16725	0.058754	0.093	0.73040	0.12	0.090202	0.040	0.590	557.0	2.1	556.79	0.50	556.73	0.21	556.82	0.21	0.05
z4	0.57	22.3	0.14	163	9184	0.058810	0.11	0.7313	0.14	0.090230	0.035	0.700	559.1	2.6	557.33	0.61	556.90	0.19	556.98	0.19	0.30
_z5	0.61	19.6	0.17	115	6413	0.058778	0.13	0.7305	0.15	0.090173	0.041	0.582	557.9	2.9	556.83	0.65	556.56	0.22	556.64	0.22	0.25
z7	0.61	41.0	0.10	392	21892	0.058752	0.065	0.73010	0.11	0.090168	0.046	0.817	556.9	1.6	556.61	0.45	556.53	0.25	556.61	0.25	0.07

All five analysed zircons from bentonite B1 (Fig. 7) are concordant, yielding a statistically equivalent weighted mean ^206^Pb/^238^U age of 556.78 ± 0.10/0.18/0.62 Ma (MSWD = 2.2, n = 5).

## Discussion

**Figure 8:**
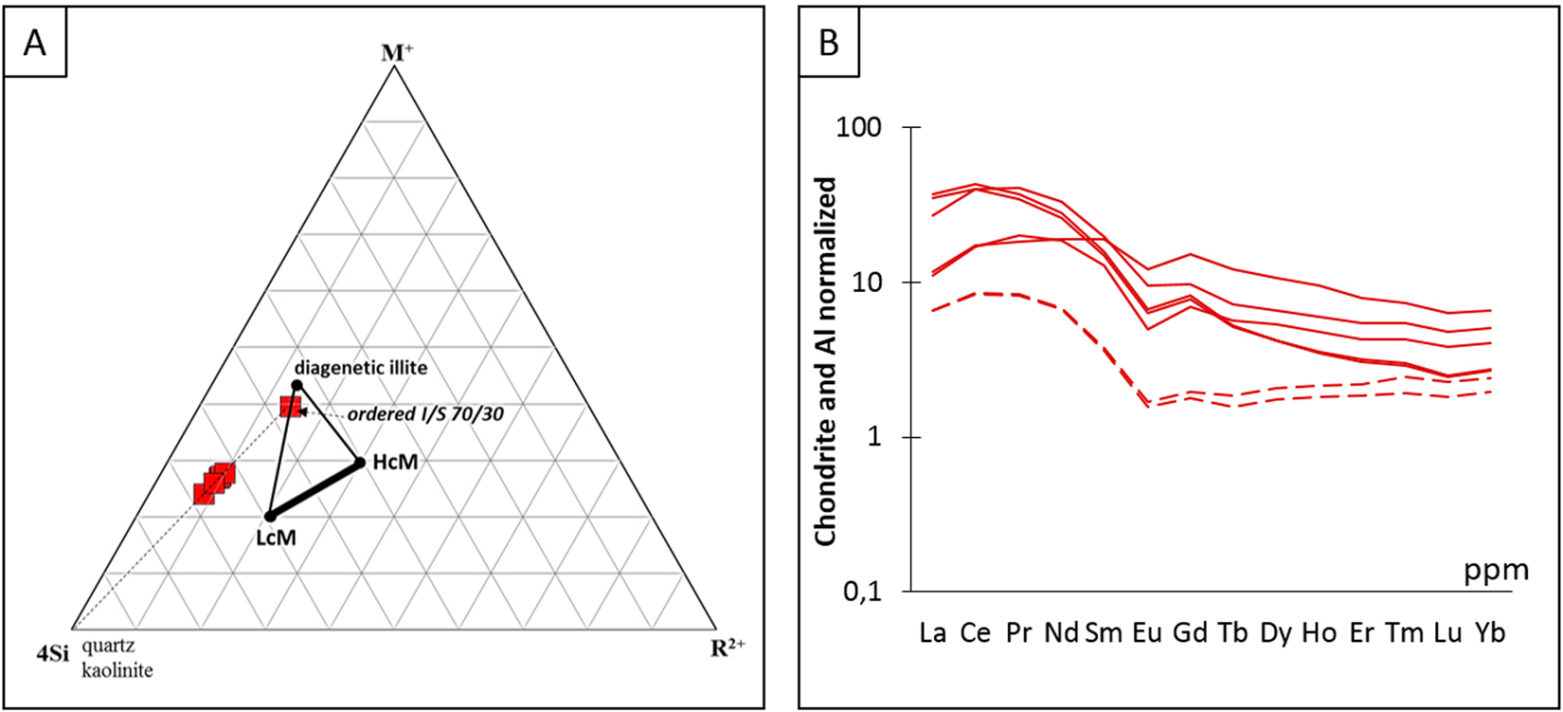
(**A**) Major element compositions plotted in 4Si-M^+^-R^2+^ system showing a proximity of bentonite to montmorillonite field; (**B**) REE distribution of altered volcanoclastic deposits of Podolya after chondrite- and Al-normalizations demonstrating similar element distribution characteristics for each K-bentonite bed.

Supported by our field observations, the mineralogical and geochemical analyses of the four Neoproterozoic clayey beds of the Mohylivska (FM) and Yarishyvska (FY) Formations sampled in the Podolya Basin show that these levels derive from volcanic ash deposits altered into K-bentonites. The difference in illite layer proportions in I-S mixed layers of investigated bentonite beds (Supplementary Fig. 2) (70/30) and the host sediments (85/15) is identical to the diagenetic conditions reported in other sedimentary basins (e.g., the Slovak basin^47^). Illitization kinetics seems to be slower in bentonites than in detrital sediments. It is noticeable here that the chemical composition of the diagenetic mineral assemblage in the bentonite beds is consistent with a mixture of quartz, kaolinite and a single I-S phase (Fig. 8a).

The distribution of REE elements in the bentonite beds after chondrite- and Al-normalizations shows a significant similarity, which confirm the common source of all these altered igneous materials (Fig. 8b). The composition of initial eruptive material corresponds to alkaline rhyolite-type, e.g., from a calc-alkaline magmatism series related to an arc setting. Moreover, the B4 K-bentonite layer contains zircon grains with sharp edges, which exclude the possibility of secondary transport and reworking. Therefore, our zircon-based chronological assessment of the bed can be considered as the absolute age of the ash deposition and thus be used to constrain the chronological span of the Ediacaran fossils of the Podolya Basin.

Only one previous age measure of 553 Ma exists for the Podolya Basin^28^. However, so far it has been difficult to assess its degree of accuracy because the analyses were not accompanied by a lithostratigraphical characterisation of the context, nor by mineralogical, petrographical or geochemical data. The age of the bentonite B4 bed, which is stratigraphically 40–45 m above the bentonite B1 from the Mohylivska (FM) Formation, is 555.4 ± 2.9 Ma. The age of the uppermost bentonite B1 of FM is 556.78 ± 0.10/0.18/0.62 Ma. On the basis of these two dates, we can now confidently infer that the transition between the Mohylivska and the Yarishivska Formations is within the uncertainty of the LA-ICP-MS method and can be constrained between 556.78 Ma and 555.4 Ma.

During this relatively short period of ~1.38 Ma, the Ediacaran macrobiota distribution at the Neoproterozoic Podolya Basin experienced notable changes: from abundant, spread and characterized by a variety of morphotypes (e.g., *Nemiana simplex, Beltanelliformis, Cyclomedusa plana, Intrites punctatus*), it experienced progressive depletion, until its disappearance in the fossil record. Perhaps, such preservation bias of the Ediacaran fauna can be also associated with changes occurred in the sedimentary regimes determining less favourable conservation conditions.

The Ediacaran biota from the Podolya Basin has great potential relevance in terms of litho-biostratigraphic correlation with other occurrences observed in similar siliciclastic successions worldwide variably rich in volcanic ash deposits representing potential sources of nutrients affecting bioproductivity^48–51^. Such Ediacaran-type assemblages have been preserved in similar geodynamic^52^ and sedimentary contexts also in several geographical areas of the ancient microcontinent Baltica, notably the White Sea, Urals, or its neighbourhood.

Following the prevalent morphotypes in the preserved fossil record and the progressive integration of refined U-Pb dates of the Ediacaran facies, three time-related major assemblages were established for this biota: the “Avalon” (579–559 Ma), the “White Sea” (558–550 Ma) and the “Nama” (549–542 Ma)^14,18^ assemblages. In this context, the Ediacaran macrofossils of the Podolya Basin^27,30^ distinctly exhibit closer affinities with some morphotypes forming the Avalon assemblage^12,13^. Indeed, one of the most common representatives in both assemblages is the taxon *Intrites punctatus*^12^ (Fig. 4B), which is typical of an early stage of the Ediacaran development because devoid of any complex structural feature. Conversely, more complex forms, such as *Kimberella, Charnia, Ovatoscutum*, have been reported in the assemblage from the Zimnie Gori Section of the White Sea^53^. Accordingly, the differences in composition observed between the Ediacaran fossil remains represented in the Podolya Basin and in the Zimnie Gori Sections could be related to the rifting dynamics of the Rodinia-Pannotia supercontinent occurred across the Ediacaran-Paleozoic^52^.

Based on its morphological similarities with the Avalon assemblage and on its constrained chronological context, the Ediacaran macrobiota of the FM Formation of the Ukrainian Podolya Basin can be now directly compared to similar penecontemporary evidence from the Chernyi Kamen Formation, in Central Urals (Russia), dated to 557 ± 13 Ma^28,54^, and to the Welsh Wrekin Terrane (southern UK), dated to 559.3 ± 2 Ma^12,55^, the latter forming part of the East-Avalonia terrane close to Baltica around the end of the Neoproterozoic^12,13^ (Table 4).

**Table 4:** Summary of different ages obtained by previous works from several Neoproterozoic localities (*data from this study).

Locality	Type of dating materials	Age	Dating method	Type of enclosing facies	Biota (prevailing morphotypes)
**Podolya (Ukraine)**	Bentonite*	555,4 ± 2,9 Ma	LA-ICP-MS	Clayey siltstone with sandy lenses	*Nemiana simplex, Beltanelliformis, Cyclomedusaplana, Intritespunctatus*
	Bentonite*	556,82 ± 0,2 Ma	ID-TIMS	Interbedded fine-grained sandstone and sandy siltstone	
	Volcanic tuff^27^	553 Ma	Methodology non precised by authors^27^	Not precised by authors^27^	
**Wrekin Terrane (Wales)**	Tuff^12,49^	559,3 ± 2 Ma	SHRIMP	Interbedded siltstone and medium-grained sandstone	*Intritespunctatus, Beltanelliformi, Medusinitess*
**Ural (Russia)**	Volcanic tuff^24,48^	557 ± 13 Ma	SHRIMP	siltstones	*PalaeopascichnusPalij, CyclomedusaSprigg*
**White Sea (Russia)**	Volcanic ash^50^	555,3 ± 0,3 Ma	ID-TIMS	claystones	*Kimberella, Charnia, Ovatoscutum, Staurinidia*

## Conclusion

Present new U-Pb-based dates of zircons from two (B1 and B4 K-bentonites) bentonite layers of the Mohylivska and Yarishyvska Formations of the Podolya Basin constrain the local presence of the Ediacaran biota between 556.78 ± 0.18 Ma and 555.4 ± 2.9 Ma. From a phylogenetic point of view, the Podolya’s macrofauna consists of relatively primitive forms compared to those from the White Sea assemblage where, approximately at the same time (555.3 ± 0.3 Ma), bilaterians were identified^53^. Paleogeographic reconstructions of the late Neoproterozoic indicate a position of the paleocontinent Baltica in the immediate vicinity of the Avalon system^12–14^, which may explain why the Ediacaran fossils from the Podolya Basin display a closer resemblance with some Avalon-type macro-organisms. Accordingly, the new chronostratigraphic evidence weakens the traditional hypothesis of a temporal sequence of the Avalon and White Sea assemblages^11^ and rather points to the need of additional research for furtherly refining the paleogeographic scenarios at the end of the Neoproterozoic Era.

## Methods

Mineralogical compositions of the bulk bentonite samples and their clay fraction (<2 µm) have been compared to that of under- and overlying deposits using X-ray diffraction. Bulk analyses were carried out on the material previously crushed and sieved at 50 μm and mounted in randomly ordered powder mode in order to characterize (hkl) reflections. The <2 μm fractions have been separated by sedimentation after dispersion and centrifugation at 20 °C −1000 rpm during 120 s using a JOUAN GR 422 centrifuge. After drying, 15 mg of clay were dispersed in 1.5 mL of osmosed water. The solution was deposited on a glass slide to study position of (00 *l*) reflections in different states: air-dried and ethylene-glycol solvatation^56,57^. The obtained diffraction profiles were compared to ones calculated with NEWMOD software for illite-smectite mixed layer minerals.

The major, trace and rare earth elements (REE) were analyzed by spectrometry of inductively coupled plasma emission (ICP) at the department of Rocks and Minerals analysis (SARM) in the Petrographic and Geochemical Research Center (CRPG) of Nancy, France.

Bentonites B1 and B4 were subjected to radioisotopic dating using single zircon grains that were separated after dispersion of 200 g of bentonite in sodium solution (1 N NaCl).

U-Th-Pb isotope data from bentonite B4 were measured by laser ablation inductively coupled mass spectrometry (LA-ICP-MS) at the Laboratoire Magmas & Volcans of Clermont-Ferrand, France. Zircons were ablated using a Resonetics Resolution M-50 laser system operating at a wavelength of 193 nm coupled to a Thermo Element XR ICP-MS. Helium carrier gas was supplemented with N_2_ prior to mixing with Ar for sensitivity enhancement^58,59^. The laser was operated with a spot diameter of 20 µm, a repetition rate of 3 Hz, and a fluence of 2.5 J/cm^2^. Instrumental operating conditions and data acquisition parameters are basically similar to that reported in Hurai *et al*.^44^ and Moyen *et al*.^59^. Reduction of raw data was carried out using the GLITTER® software package of Macquarie Research Ltd^60^. Isotope ratios were corrected for laser-induced and instrumental mass fractionation via sample-standard bracketing using the GJ-1 zircon (^206^Pb/^238^U age of 601 Ma^61^). Data were not corrected for common Pb. ^207^Pb/^206^Pb vs. The ^238^U/^206^Pb diagram was generated using the Isoplot/Ex v. 2.49 software of Ludwig^62^. Error ellipses for each point are shown at the 2σ level and incorporate both internal and external uncertainties. Data points were pooled to calculate a date and associated 2σ error. The 91500 zircon reference material −1065 Ma^62^ - was analyzed along with the samples to independently monitor the external precision and accuracy of the measurements. The Concordia age for 132 analyses of 91500 conducted over the course of the study was 1063.9 ± 2.4 Ma (2σ including decay constant errors). All data are reported in Table 1.

Zircons from bentonite B1 were analyzed by high precision U-Pb Chemical Abrasion Isotope Dilution Thermal Ionisation Mass-Spectrometry (CA-ID-TIMS) at University of Geneva, Switzerland. After initial dissolution of the clay minerals, the remaining sample was subjected to methylene iodide heavy liquid separation. The retrieved zircons were predominantly long prismatic, with clear pyramids and sharp prisms. Selected grains were subjected to annealing and chemical abrasion (CA) following Mattinson^63^. The annealing conditions were 900 °C for 48 h, while the chemical abrasion was done by placing each individual zircon into a pre-cleaned Savillex capsule with HF + trace HNO_3_ at 210 °C for up to 13 h in a Parr bomb. After the partial dissolution step, each zircon together with the leachate was again transferred into a 3 ml screw-top Savillex vial. The leachate was completely pipetted out and the remaining zircons were rinsed in ultrapure water and then fluxed for several hours in 6 N HCl on a hotplate at a temperature of ca. 80 °C. After removal of the acid, the zircon fragments were again rinsed several times in ultra-pure water and 7 N HNO_3_ in an ultrasonic bath. Each single zircon grain was loaded for dissolution into pre-cleaned Savillex capsules, spiked with approximately 5 mg of the EARTHTIME ^202^Pb-^205^Pb-^233^U-^235^U tracer solution^64^. The isotopic analyses were performed at University of Geneva on a TRITON mass spectrometer equipped with a MasCom discrete dynode electron multiplier. The linearity of the multiplier was calibrated using U500, Sr SRM987, and Pb SRM982 and SRM983 solutions. The deadtime for the SEM was determined to be constant at 22.5 ns for up to 1.3 Mcps and at a Faraday/SEM yield between 93–94%. Lead and uranium isotopic fractionation were corrected using the ratios ^202^Pb/^205^Pb (0.9992391 ± 0.0265%, 1σ) and ^233^U/^235^U (0.99506 ± 0.01%, 1σ) of the double spike solution. The average Pb and U fractionation factors determined were 0.13 ± 0.02%/amu and 0.09 ± 0.02%/amu (1σ), respectively. Pb and U (as UO_2_) isotope compositions were measured on the electron multiplier. Isobaric interference of ^233^U^18^O^16^O on ^235^U^16^O^16^O was corrected using an ^18^O/^16^O ratio of 0.00205. The measured uranium isotopic ratios were corrected assuming a sample ^238^U/^235^U ratio of 137.818 ± 0.045^65^. All common Pb in the zircon analyses was attributed to the procedural blank with the following lead isotopic composition: ^206^Pb/^204^Pb = 17.62 ± 2.09, ^207^Pb/^204^Pb = 14.73 ± 3.06, ^208^Pb/^204^Pb = 35.77 ± 2.99 (1-sigma %). The initial statistics, data reduction and age calculation were done using the TRIPOLI and Redux software^66^. All ^206^Pb/^238^U and ^207^Pb/^206^Pb ratios were corrected for initial disequilibrium in ^230^Th/^238^U using Th/U (magma) assuming Th/U of the magma = 3.5. The accuracy of the measured data was assessed by repeated analysis of the 100 Ma synthetic solution^65^ and the international R33 zircon standard^67^, which was pre-treated by chemical abrasion. Both yielded an internal reproducibility in ^206^Pb/^238^U dates of better than 0.05%. All uncertainties reported are at the 2 sigma level, following x/y/z systematic of Schoene *et al*.^66^. All data are reported in the Table 2 with internal errors only, including counting statistics, uncertainties in correcting for mass discrimination, and the uncertainty in the common (blank) Pb composition. The MSWD value of weighted mean from the sample is within the range of acceptable values at 95% confidence level and for (n - 1) degrees of freedom.

## Supplementary information

Supplementary information
