# Supplementary material for: Precise U-Pb age constrains on the Ediacaran biota in Podolia, East European Platform, Ukraine
Source: Sci Rep. 2019 Feb 8;9:1675. doi: 10.1038/s41598-018-38448-9 (PMC6368556; doi:10.1038/s41598-018-38448-9)
Supplement: Supplementary file 1 — Supplementary information [file 41598_2018_38448_MOESM1_ESM.docx]

**Precise U-Pb age constrains on the Ediacaran biota in Podolia, East European Platform, Ukraine**

Y. Soldatenko^1,2^, A. El Albani^1*^, M. Ruzina^2^, C. Fontaine^1^, V. Nesterovsky^3^, J.-L. Paquette^4^, A. Meunier^1^ & M. Ovtcharova^5^

^1^Université de Poitiers, Institut de Chimie des Milieux et Matériaux de Poitiers, CNRS UMR 7285, 86073 Poitiers, France. ^2^Department of Geological Prospection, National Mining University of Ukraine, 49066 Dnipro, Ukraine. ^3^Department of Geology, National Kiev University of Taras Shevschenko, Kiev, Ukraine. ^4^Laboratoire Magmas et Volcans, Université Clermont-Auvergne-CNRS-IRD-OPGC, 63000 Clermont-Ferrand, France. ^5^Department of Earth Sciences, University of Geneva, 13 rue des Maraîchers, 1205 Genève, Switzerland.

**Supplementary Information**

**Lithostratigraphy**

**
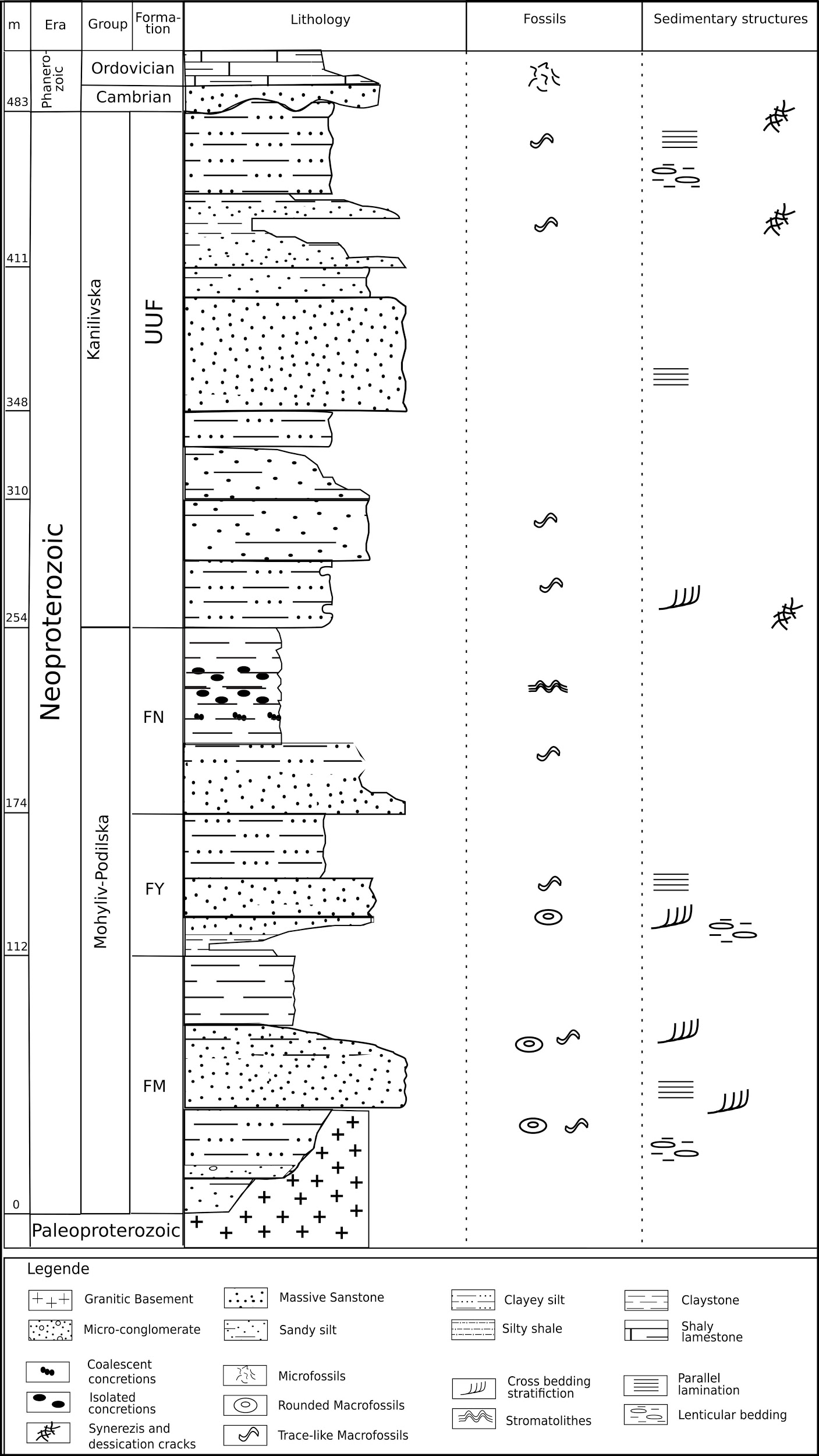
**

**Supplementary Fig. 1.** Stratigraphic Log (after^27-29^) showing the succession in the late Neoproterozoic of the Podolya Basin, southwestern Ukraine. FM: abbreviation for Mohylivska Formation; FY: abbreviation for Yarishyvska Formation; FN: abbreviation for Nagoryanska Formation; UUF: abbreviation for Upper Undifferentiated Formations (according to the local stratigraphic nomenclature).

**Mineralogy.** The mineralogy of the bentonite beds is very different from that of the lower and the upper adjacent sediments. This is typically shown by the B4 sample compared to clayey and silts of the Yarishyvska Formation (FY) (Supplementary Fig. 2). The bulk mineralogy of the host sediments is dominated by detrital quartz, K-feldspar and plagioclase, which are absent (feldspars) or discrete (quartz) in the B4 bed (Supplementary Fig. 2A). Furthermore, the clay fractions (<2 µm) are also totally different (Supplementary Fig. 2B). Indeed, the typical clay mineralogy of the FY deposits is mainly composed of illite/smectite mixed-layer minerals (I:S ratio of 85:15), kaolinite, and traces of illite or white mica (Supplementary Fig. 2B). Interstratified illite/chlorite have been detected in some silts of the siliciclastic deposits, with I:C ratio close to 50:50. The clay mineralogy of the B4 sample is largely composed of R1 illite/smectite mixed-layer, with I:S ratio of 70:30 associated with well-crystallized kaolinite. As a result, the B4 sample appears as an almost mono-mineral clay deposit composed of an ordered illite/smectite mixed-layer, which contrasts with the illite-richer mixed-layers of the host terrigenous sediments. Finally, the sharp contact with the underlying deposits and the absence of terrigenious material indicate that the bentonite layer is not of detrital origin, but represents an in situ transformed ash deposit. As suggested by the absence of zeolites^1,2,3^, ash alteration took place under marine conditions.


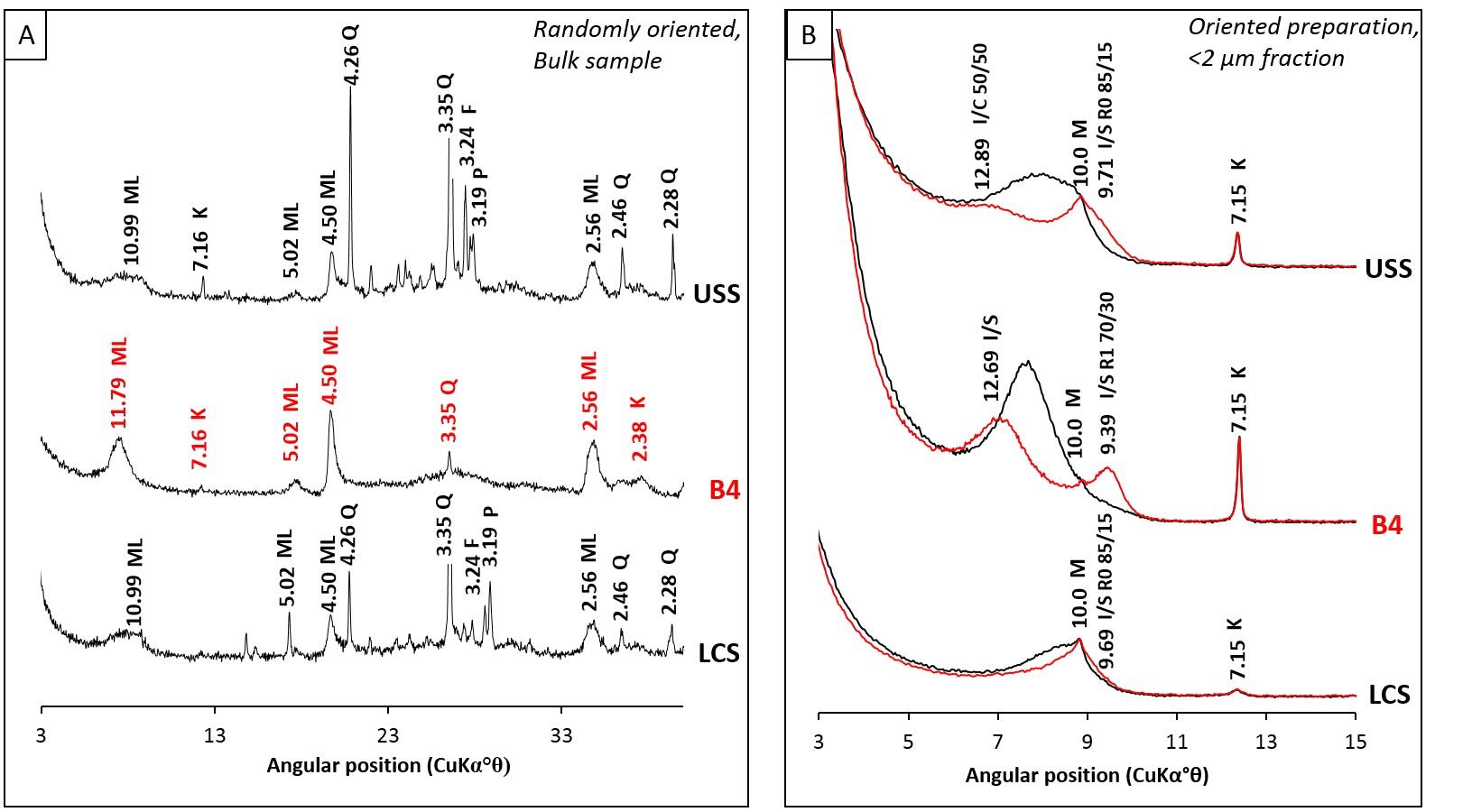


**Supplementary Fig. 2.** Diffractogram patterns of: A) Bulk sample (random prepared powder) of lower clayey silts (LCS) and upper sandy silts (USS) and B4 bentonite bed of the Yarishyvska Formation (FY); B) <2 µm fraction (oriented preparation) of B4 bentonite, LCS and USS samples in air dried (black) and ethylene glycol solvated treatment (red). Identified minerals: (Ca) calcite; (F) K-feldspar; (I/C) illite-chlorite mixed-layer; (I/S) illite-smectite mixed-layer; (K) kaolinite; (M) mica; (ML) undifferentiated mixed-layers; (P) plagioclase; (Q) quartz.

**References**

1. Hay, R. L. Stratigraphy and zeolitic diagenesis of the John Day Formation of Oregon. California Univ. Publ. Geol. Sci. **42**, 199-262 (1963).
2. Hess, P. C. Phase equilibria of some minerals in the K_2_O-Na_2_O-AI_2_O_3_-SiO_2_-H_2_O system at 25° C and 1 atmosphere. *Amer. J. Sci*. **264**, 289-309 (1966).
3. Sheppard, R. A. & Gude, A. J. *Distribution and Genesis of Authigenic Silicate Minerals in Tuffs of Pleistocene Lake Tecopa, Inyo County, California*. (*U.S. Geol. Surv., Prof. Pap*. **597**, United States Government Printing Office, 1968).
